# Both people living in the COVID-19 epicenter and those who have recently left are at a higher risk of loneliness

**DOI:** 10.1038/s41598-023-47140-6

**Published:** 2023-11-30

**Authors:** Yan-Min Xu, Ming-Fang Wang, Bao-Liang Zhong

**Affiliations:** 1 Department of Psychiatry, Wuhan Mental Health Center, Wuhan, Hubei Province China; 2 Center for Psychological Consultation and Therapy, Wuhan Hospital for Psychotherapy, Wuhan, Hubei Province China; 3 Department of Clinical Nutrition, Beijing Hospital, National Center of Gerontology, Institute of Geriatric Medicine, Chinese Academy of Medical Sciences, Beijing, China

**Keywords:** Psychology, Diseases, Health care

## Abstract

There is little empirical data on the heightened risk of loneliness among individuals residing in the COVID-19 epicenter or those who have recently left. This study compared the risk of loneliness in individuals residing in Wuhan, the COVID-19 epicenter in China, and those who had recently left during the initial outbreak period to those living in non-epicenter regions. During the COVID-19 outbreak in China in 2020, three samples were obtained using snowball sampling. The samples included 2882 epicenter residents, 1028 left residents, and 2963 non-epicenter residents. Loneliness was assessed using the six-item De Jong Gierveld Loneliness Scale, with a score of two or more indicating the presence of loneliness. 53.5% and 55.2% of epicenter and recently left residents, respectively, experienced loneliness, which was significantly higher than the 43.9% observed in non-epicenter residents. After adjusting for covariates, the risk of loneliness remained statistically significant for both epicenter and left residents (OR = 1.35, *P* < 0.001 and OR = 1.20, *P* = 0.017, respectively), when compared to non-epicenter residents. Individuals residing in the COVID-19 epicenter, as well as those who have recently left, are at a heightened risk of loneliness during the outbreak. These individuals need psychosocial support to mitigate their risk of loneliness and promote their psychological wellbeing.

## Introduction

The COVID-19 pandemic has brought about significant changes in our daily lives, including quarantine and distancing measures, which have resulted in an increased risk of social isolation and loneliness^1–5^. People residing in COVID-19 epicenters, where the prevalence of SARS-CoV-2 is highest, may be at a higher risk of experiencing loneliness as a result of the stringent lockdown measures and restrictions on social gatherings mandated by the authorities, compared to those residing outside these epicenters. In the literature, several studies have reported a higher risk of mental health problems in people living within epicenters compared to those outside, such as depressive symptoms, insomnia symptoms, and low levels of affective and cognitive wellbeing^6–10^. However, there is still a lack of empirical data on the increased risk of loneliness in individuals residing in COVID-19 epicenters.

Because of the risk of spreading SARS-CoV-2, individuals who live in or have visited COVID-19 epicenters and recently traveled to other locations are generally required to quarantine. They may also be separated from family members who are currently under lockdown in those epicenters^10^. In addition, people living in non-epicenters are more likely to keep their distance from individuals who are from COVID-19 epicenters due to concerns about contracting SARS-CoV-2^11^. Despite this, there is very limited empirical evidence regarding the heightened risk of loneliness in persons who have recently left COVID-19 epicenters.

To the best of our knowledge, only one prior study conducted by our colleagues has assessed the risk of loneliness among individuals based on their locations during the COVID-19 outbreak in China^3^. This study observed statistically higher prevalence rates of loneliness in two groups: residents living within the COVID-19 epicenter (25.3%) and individuals who recently relocated to the epicenter but currently reside in other parts of China (27.2%), compared to residents in other regions of China (23.0%). However, this study did not primarily focus on the differences in the risk of loneliness among subpopulations. The significant subpopulation differences in the risk of loneliness also disappeared in the subsequent multiple regression analysis. It's worth noting that the prior study’s measure of loneliness is potentially problematic, as it employed a single-item measure that has been criticized for its tendency to underreport the true feelings of loneliness in population-based surveys^12^.

From January 23 to April 8, 2020, in order to prevent the spread of SARS-CoV-2 to other parts of China, Wuhan shut down all inbound and outbound transportation, and implemented a stay-at-home policy for the nine million residents who remained in the city^12,13^. Due to the Spring Festival holiday and the panic caused by the outbreak, approximately five million people left Wuhan in the weeks before the lockdown^13^. In order to improve the planning of mental health services during the COVID-19 pandemic, it is crucial to identify subpopulations that are at a higher risk of experiencing loneliness. Therefore, this study compared the risk of loneliness in individuals residing in Wuhan, the COVID-19 epicenter in China, and those who had recently left during the initial outbreak period to those living in non-epicenter regions.

Loneliness is typically defined as a negative subjective feeling resulting from a lack of satisfactory quality and/or quantity in a Person’s social connections^14,15^. It is an important indicator of well-being and has been associated with an increased risk of various physical and mental morbidities as well as mortality^16–18^. In the context of the pandemic, Choi and colleagues argued that the COVID-19 pandemic and its associated restrictions have limited opportunities for social interactions, reduced necessary social support, and disrupted the quality of relationships. This has resulted in a discrepancy between an individual's actual social connections and his/her desired connections^19^. The pandemic has directly restricted people’s social interactions^20^. However, this negative impact varies among different subpopulations, being more pronounced among individuals who were quarantined in the epicenter or who have recently relocated. Therefore, we hypothesized that individuals residing in Wuhan, China, and those who had recently left during the initial outbreak period would face a higher risk of loneliness compared to those living in non-epicenter regions.

## Results

The analysis included 2882 epicenter residents, 1028 left residents, and 2963 non-epicenter residents. The epicenter residents were found to be statistically older (36.9 ± 11.1 vs. 34.7 ± 9.9 years, *P* < 0.001) than non-epicenter residents, while the left residents were statistically younger (29.5 ± 9.2 vs. 34.7 ± 9.9 years, *P* < 0.001). Additionally, both epicenter and left residents had a statistically higher percentage of male participants compared to non-epicenter residents (40.7%and 45.8% vs. 35.0%, *P* < 0.001). The sociodemographic characteristics and SARS-CoV-2 infection status of the families and acquaintances of the three subpopulations are presented in Table 1 with detailed data.

**Table 1:** Sociodemographic characteristics and SARS-CoV-2 infection status of the participants’ families and acquaintances of epicenter residents, left residents, and non-epicenter residents.

Factor	Level	Residents in the epicenter (n = 2882)	Residents who left the epicenter (n = 1028)	Residents in non-epicenter provinces (n = 2963)	χ2	*P*
Sex	Male	1174 (40.7)	471 (45.8)	1036 (35.0)		
	Female	1708 (59.3)	557 (54.2)	1927 (65.0)	44.01	< 0.001
Age-group (years)	18–30	784 (27.2)	611 (59.4)	1219 (41.1)		
	31–40	1053 (36.5)	290 (28.2)	1011 (34.1)		
	41 +	1045 (36.3)	127 (12.4)	733 (24.7)	408.477	< 0.001
Marital status	Married, remarried, or cohabiting	1856 (64.4)	396 (38.5)	1592 (53.7)		
	Never married	904 (31.4)	616 (59.9)	1266 (42.7)		
	Separated, divorced, or widowed	122 (4.2)	16 (1.6)	105 (3.5)	269.182	< 0.001
Educational attainment	Middle school or lower	650 (22.6)	230 (22.4)	296 (10.0)		
	Associate	626 (21.7)	232 (22.6)	430 (14.5)		
	Bachelor	1224 (42.5)	355 (34.5)	1467 (49.5)		
	Master or higher	382 (13.3)	211 (20.5)	770 (26.0)	366.496	< 0.001
Employment status	Employed	2300 (79.8)	557 (54.2)	2185 (73.7)		
	Unemployed	582 (20.2)	471 (45.8)	778 (26.3)	254.937	< 0.001
SARS-CoV-2 infection of family members or close friends	No	2797 (97.1)	1022 (99.4)	2597 (99.8)		
	Yes	85 (2.9)	6 (0.6)	6 (0.2)	85.175	< 0.001
SARS-CoV-2 infection of colleagues, classmates, or other friends	No	2248 (78.0)	946 (92.0)	2925 (98.7)		
	Yes	634 (22.0)	82 (8.0)	38 (1.3)	653.04	< 0.001
Loneliness	No	1340 (46.5)	461 (44.8)	1663 (56.1)		
	Yes	1542 (53.5)	567 (55.2)	1300 (43.9)	69.124	< 0.001

Both the epicenter and left residents showed a significantly higher prevalence of loneliness compared to non-epicenter residents, with rates of 53.5% and 55.2% versus 43.9%, respectively (*P* < 0.001), as presented in Table 1. After controlling for covariates, the risk of loneliness remained statistically significant for both epicenter and left residents (OR = 1.35, *P* < 0.001 and OR = 1.20, *P* = 0.017, respectively) compared to non-epicenter residents, as shown in Table 2.

**Table 2:** The relative risk of loneliness in epicenter residents and left residents compared to non-epicenter residents, adjusting for covariates by using multiple logistic regression analysis.

Factor	Level	OR (95%CI)	*P*
Category of residents	Residents in the epicenter	1.35 (1.20, 1.51)	< 0.001
	Residents who left the epicenter	1.20 (1.03, 1.40)	0.017
	Residents in non-epicenter provinces	1	
Sex	Male	1.35 (1.22, 1.49)	< 0.001
	Female	1	
Age-group (years)	18–30	1.76 (1.46, 2.12)	< 0.001
	31–40	1.65 (1.45, 1.88)	< 0.001
	41+	1	
Marital status	Never married	1.35 (1.16, 1.58)	< 0.001
	Separated, divorced, or widowed	1.64 (1.25, 2.14)	< 0.001
	Married, remarried, or cohabiting	1	
Educational attainment	Middle school or lower	2.32 (1.95, 2.76)	< 0.001
	Associate	1.991.69, 2.33)	< 0.001
	Bachelor	1.45 (1.27, 1.66)	< 0.001
	Master or higher	1	
Employment status	Unemployed	1.12 (0.98, 1.28)	0.110
	Employed	1	
SARS-CoV-2 infection of family members or close friends	Yes	1.96 (1.26, 3.05)	0.003
	No	1	
SARS-CoV-2 infection of colleagues, classmates, or other friends	Yes	1.28 (1.08, 1.52)	0.004
	No	1	

## Discussion

To the best of our knowledge, this is the first study to compare the likelihood of experiencing loneliness among residents who underwent lockdowns in the epicenter, recently departed from the epicenter, and were situated in non-epicenter regions. The primary findings of this study indicate a prevalence of loneliness at 53.5% and 55.2% in epicenter and recently departed residents, respectively, which is higher than the 43.9% found in non-epicenter residents. Moreover, the independent relative risk of loneliness in both epicenter and recently departed residents was significantly greater than that of non-epicenter residents, with ORs of 1.35 and 1.20, respectively. Our study has addressed a gap in the current literature by demonstrating a significant increase in the risk of loneliness among epicenter and recently left residents, with a 35% and 20% greater likelihood, respectively, when compared to non-epicenter residents. These findings identify specific subpopulations that would benefit from targeted psychosocial services to reduce the risk of loneliness among those affected by outbreaks of COVID-19 and other infectious diseases in the future.

The relative risk of experiencing loneliness in the three subpopulations is comparable to the relative risk of developing mental health problems in these subpopulations, which were impacted differently by the COVID-19 outbreak^10^. Specifically, the ORs for depressive symptoms among individuals residing in the epicenter, as well as among migrants from the epicenter, in comparison to those living in other areas were 1.61 and 1.56, respectively^10^. As a result of the strict mass quarantine imposed in Wuhan, it is expected that the residents in the epicenter face the greatest risk of loneliness. Even those who have recently left Wuhan are required to undergo medical observation and isolation at their destination sites, and they are unable to reunite with their families during the Spring Festival. It is also possible that some of these individuals may experience high levels of social stigma and discrimination, given the labels of “potential SARS-CoV-2 carriers”. These factors may contribute to the elevated risk of loneliness experienced by these left residents. The heightened risk of loneliness among epicenter residents and left residents, in comparison to non-epicenter residents, could be attributed to the increased levels of social isolation experienced by the two groups as a result of the pandemic and the stringent containment measures that were implemented, despite the nationwide containment measures being in place.

The primary limitation of this study is the limited representativeness of the sample, which was a convenience sample of individuals residing in the COVID-19 epicenter, those who recently left, and individuals living in non-epicenter regions. In fact, the majority of the participants were social media users. A potential significant limitation of snowball sampling via social media platforms is that participants may invite others with similar values or personalities to join the study. In our case, we did not assess the homophily among participants, so we are unable to account for this potential bias when determining the risk of loneliness among subpopulations. Moreover, the experience of loneliness among Chinese individuals during the outbreak may differ from that in other countries due to differences in socio-economic and cultural contexts, as well as varying COVID-19 containment strategies. This limited sample may not be entirely representative of the broader population, which could impact the generalizability of the study's findings. Second, we did not thoroughly evaluate the specific characteristics of relocated residents, such as the duration of migration from the epicenter and the geographical distances between the epicenter and their destination locations. Additionally, it is plausible that quarantine in an unfamiliar environment could heighten feelings of loneliness. However, we did not gather corresponding data, which made it challenging to provide comprehensive explanations for the increased risk of loneliness among the relocated residents. Third, we did not account for other potential factors, such as major medical conditions, depression, and anxiety, in our multiple logistic regression analysis to assess the risk of loneliness across the three subpopulations. The reason for the omission of negative emotions is the absence of evidence regarding the chronological order between depression, anxiety, and loneliness. Considering the significant impact of the pandemic and its associated restriction measures, we are inclined to believe that depression, anxiety, and loneliness are outcomes of the pandemic that occurred concurrently after the outbreak. Therefore, we argue that loneliness is less likely a consequence of depression and anxiety in our study. Fourth, while qualitative data can be useful in uncovering the underlying causes of the elevated risk of loneliness and developing more comprehensive psychosocial interventions, our study did not include any qualitative research to investigate this matter further.

Notwithstanding these limitations, the elevated risk of loneliness observed in individuals residing in the COVID-19 epicenter, as well as those who have recently left, highlights the importance of prioritizing mental health services for these subpopulations during the outbreak. To improve the psychological wellbeing of affected adults during this period, mental health and social workers should consider implementing periodic screenings for loneliness and other mental health problems in both subpopulations and providing timely psychosocial support services to those in need.

## Methods

### Participants

This comparative study was a secondary analysis of data from a large-scale online cross-sectional survey conducted between January 27 and February 10, 2020^10^^,^^21^. This survey duration coincided with the early stage of the rapid spread of the COVID-19 epidemic and the lockdown of Wuhan in China. Due to logistical challenges during the emergency period and concerns regarding the transmission of SARS-CoV-2, it was not feasible to conduct household-based surveys. Therefore, the questionnaire data was collected online using a snowball sampling method. Participants were recruited through targeted networks of individuals residing in Wuhan and other cities in China. An invitation letter was distributed among their social networks and posted to their WeChat (similar to "WhatsApp") and Weibo (similar to “Twitter”) accounts. The invitation letter introduced the survey and included a quick response code for accessing the online questionnaire. This study enrolled Chinese adults who were 18 years of age or older, living in communities located in mainland China, willing to participate in the survey, and had no known or suspected SARS-CoV-2 infection. The details of the sampling and participant recruitment have been previously published elsewhere^3,10,21,22^.

The survey protocol and informed consent procedures were approved by the Medical Ethics Committee at Wuhan Mental Health Center. Prior to self-administering the questionnaire, we obtained informed consent from all participants by having them sign the consent form electronically. The protocol and methods were conducted in accordance with the Declaration of Helsinki and relevant ethical guidelines and regulations in China.

### Procedures and instruments

The survey questionnaire was completed anonymously and online, using a self-administration format. The sociodemographic variables in the questionnaire included sex, age, marital status, education, and employment status. Participants were asked to report their current place of residence, and whether they had lived, worked, studied, or visited Wuhan during the two months preceding the survey if they were not residing in Wuhan. Based on this information, participants were categorized into three groups: those residing in Wuhan (“epicenter residents”), those who had left Wuhan (“left residents”), and those residing in non-epicenter provinces (“non-epicenter residents”). In the current analysis, we excluded individuals residing in the surrounding areas of Wuhan in Hubei province due to the severe outbreak that originated from the epicenter.

To determine the SARS-CoV-2 infection status of the participants’ families and acquaintances, two yes–no questions were asked: “Do you have any family members or close relatives infected with SARS-CoV-2?” and “Do you have any colleagues, friends, or classmates infected with SARS-CoV-2?”.

This study used the validated Chinese version of the six-item De Jong Gierveld Loneliness Scale (DJGLS) to evaluate participants' recent experiences of loneliness^23–25^. The original DJGLS was developed by Gierveld and Tilburg, and its Chinese adaptation was cross-culturally validated by Leung and colleagues^24,26^. The Cronbach’s α coefficients for the Chinese DJGLS were 0.76 and 0.82 in the prior validation study and the current study, respectively. In the validation study, a correlation coefficient of 0.71 (*P* < 0.001) was observed between the DJGLS total score and the direct yes–no loneliness question score. In the present study, a correlation coefficient of 0.433 (*P* < 0.001) was found between the DJGLS total score and the single-item five-point Likert loneliness question score. The DJGLS total score is obtained by summing the six items, which ranges from zero to six. A higher total score indicates greater severity of loneliness symptoms. Following the algorithm recommended by Tilburg and Gierveld, a score of two or more on the six-item DJGLS suggests the presence of loneliness^27^.

### Statistical analysis

We used a chi-square test to compare categorical variables across three subpopulations. To determine the independent risk of loneliness in epicenter residents and left residents, a multiple logistic regression analysis was performed, which included subpopulation as the main predictor (with non-epicenter residents as the reference category), along with all sociodemographic variables and SARS-CoV-2 infection status of the participants’ families and acquaintances as covariates. Odds ratios (ORs) and their 95% confidence intervals (95%CIs) were calculated to assess relative risk. Statistical significance was set at *P* < 0.05 (two-tailed). All analyses were conducted using the SPSS software version 18.0 package (SPSS Inc., Chicago, IL, USA).

## Data Availability

The data that support the findings of this study are available from the corresponding author upon reasonable request.
